# Incidence and risk factors for deep venous thrombosis of lower extremity after surgical treatment of isolated patella fractures

**DOI:** 10.1186/s13018-021-02240-9

**Published:** 2021-01-28

**Authors:** Zhanchao Tan, Hongzhi Hu, Xiangtian Deng, Jian Zhu, Yanbin Zhu, Dandan Ye, Xiaodong Cheng, Yingze Zhang

**Affiliations:** 1 Department of Orthopedic Surgery, The 3rd Hospital of Hebei Medical University, NO. 139 Ziqiang Road, Shijiazhuang, 050051 Hebei People’s Republic of China; 2 Department of Orthopedics, Union Hospital of Tongji Medical College of Huazhong University of Science and Technology, Wuhan, 430022 China; 3 School of Medicine, Nankai University, Tianjin, 300071 People’s Republic of China; 4 Key Laboratory of Biomechanics of Hebei Province, Shijiazhuang, 050051 Hebei People’s Republic of China

**Keywords:** DVT, Patella fractures, Risk factors

## Abstract

**Background:**

Limited information exists on the incidence of postoperative deep venous thromboembolism (DVT) in patients with isolated patella fractures. The objective of this study was to investigate the postoperative incidence and locations of deep venous thrombosis (DVT) of the lower extremity in patients who underwent isolated patella fractures and identify the associated risk factors.

**Methods:**

Medical data of 716 hospitalized patients was collected. The patients had acute isolated patella fractures and were admitted at the 3rd Hospital of Hebei Medical University between January 1, 2016, and February 31, 2019. All patients met the inclusion criteria. Medical data was collected using the inpatient record system, which included the patient demographics, patient’s bad hobbies, comorbidities, past medical history, fracture and surgery-related factors, hematological biomarkers, total hospital stay, and preoperative stay. Doppler examination was conducted for the diagnosis of DVT. Univariate analyses and multivariate logistic regression analyses were used to identify the independent risk factors.

**Results:**

Among the 716 patients, DVT was confirmed in 29 cases, indicating an incidence of 4.1%. DVT involved bilateral limbs (injured and uninjured) in one patient (3.4%). DVT involved superficial femoral common vein in 1 case (3.4%), popliteal vein in 6 cases (20.7%), posterior tibial vein in 11 cases (37.9%), and peroneal vein in 11 cases (37.9%). The median of the interval between surgery and diagnosis of DVT was 4.0 days (range, 1.0-8.0 days). Six variables were identified to be independent risk factors for DVT which included age category (> 65 years old), OR, 4.44 (1.34-14.71); arrhythmia, OR, 4.41 (1.20-16.15); intra-operative blood loss, OR, 1.01 (1.00-1.02); preoperative stay (delay of each day), OR, 1.43 (1.15-1.78); surgical duration, OR, 1.04 (1.03-1.06); LDL-C (> 3.37 mmol/L), OR, 2.98 (1.14-7.76).

**Conclusion:**

Incidence of postoperative DVT in patients with isolated patella fractures is substantial. More attentions should be paid on postoperative DVT prophylaxis in patients with isolated patella fractures. Identification of associated risk factors can help clinicians recognize the risk population, assess the risk of DVT, and develop personalized prophylaxis strategies.

## Background

Deep vein thrombosis (DVT) is common in hospitalized patients especially those with trauma. It is an important source of morbidity and causes fatal pulmonary embolism (PE). Researchers have investigated the occurrence of DVT in patients who had undergone orthopedic major surgeries [1]. Authors have studied DVT after major orthopedic trauma such as hip fractures [2], pelvic-acetabular fractures [3], and spinal fractures [4]. The association between trauma and DVT is well recognized [5].

However, studies on the incidence of DVT in patients with isolated fractures of the lower extremities are limited. Minimal studies have focused on the thromboembolic events in femoral shaft fracture [6], tibia fracture [7], ankle fracture [8], and calcaneal fracture [9]. However, two researchers studied the incidence of DVT in isolated patella fractures [10, 11]. Patella fractures are relatively uncommon in emergency department or orthopedics department and the reported incidence was 13.5/100,000 person/years in China [12]. The risk of DVT as a complication is easily overlooked by orthopedic surgeons. This is because such fractures are relatively minor. Determining the incidence of postoperative DVT in patients with isolated patella fracture is very important. It improves orthopedic surgeons’ rational understanding of this kind of complication. Identification of associated risk factors can help clinicians recognize the risk population, assess the risk of DVT, and develop personalized prophylaxis strategies. The research was conducted to determine the incidence and location of postoperative DVT following acute isolated patella fractures. The research also identified the correlated independent risk factors.

## Methods

This was a retrospective study. This research was approved before the start of the study by the ethics committee of the institution (the 3rd Hospital of Hebei Medical University).

### Inclusion and exclusion criteria (Fig. 1)

This research consisted of hospitalized patients with isolated patella fractures admitted in the institution between January 1, 2016, and February 31, 2019. The demographic variables and clinical data were acquired from the medical records. Inclusion criteria were as follows: (a) age > 18 years old, (b) confirmed isolated patella fracture, (c) underwent operation treatment, and (d) with complete medical data. Exclusion criteria were as follows: (a) pathological fracture, (b) old fracture (treatment delayed > 3 weeks), (c) open fracture, (d) concurrent with other fractures or cerebral trauma, (e) nonsurgical treatment, (f) incomplete clinical data, (g) administration of anticoagulants on admission for treatment of other illnesses, (h) preoperative diagnosis of DVT, and (i) patients who had suffered hemorrhagic stroke.

**Fig. 1:**
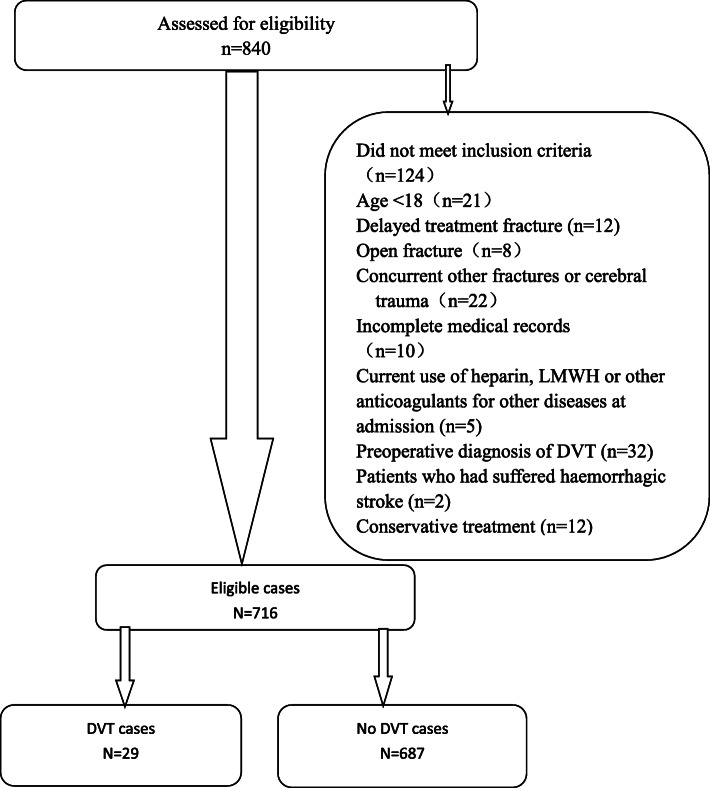
Exclusion criteria and the eligible cases included in this study

In the institution, routine anticoagulant therapy was administered to all the hospitalized patients with patella fractures (low molecular weight heparin, 2500–4100 IU once daily, subcutaneous injection). Mechanical thromboprophylaxis (ankle pump exercise) was also administered to each patient.

### Diagnosis of DVT

DVT in lower extremities was identified using duplex ultrasound scanning. The diagnostic data of DVT was obtained from the Doppler ultrasound reports.

Duplex ultrasound scanning was conducted in postoperative patients with suspected clinical symptoms such as swelling, lower limb pain, and superficial varicose veins. Deep venous obstruction or constant intraluminal filling defect was an indication of DVT diagnosed using Doppler ultrasound. Conventional scanning included the common femoral vein, superficial femoral, deep femoral vein, popliteal vein, anterior tibial vein, posterior tibial vein, and common fibular vein. Intermuscular vein thrombosis was excluded due to its less clinical significance [13].

### Data collection

Clinical data was collected using electronic medical records (EMR). The time when the postoperative duplex ultrasound examinations were conducted on patients was recorded. Data on patient demographic variables such as age, gender, BMI (body mass index), patients’ bad hobbies such as cigarette smoking and alcohol consumption were also determined. Data on comorbidities collected in this study included hypertension, diabetes mellitus, ischemic heart disease, arrhythmia, and chronic lung diseases. Data on past medical history contained history of cerebral infarction and previous surgery. Fracture-related data consisted of injury mechanism (low or high energy) and fracture type (simple or comminuted). Surgery-related data included ASA (American Society of Anesthesiologists) classification, anesthesia, surgical duration, intraoperative blood loss, and tourniquet. Preoperative stay (from injury to operation) and total hospital stay were also included in the study. BMI (kg/m^2^) was grouped into four types: normal (18.5-23.9), underweight (< 18.5), overweight (24.0-27.9), and obesity (≥ 28.0). Age was categorized into three categories: 18 and 44 years, 45 and 64 years, and > 65 years old. Low energy damage was defined as the damage caused by falling from a standing height. Other damages, such as traffic accidents and falling from a height, were defined as high-energy injuries.

The biomarkers included TP (total protein) level, ALB (albumin) level, FBG (fasting blood glucose) level, RBC (red blood cell) count, WBC (white blood cell) count, NEUT (neutrophile) count, LYM (lymphocyte) count, HGB (hemoglobin) level, HCT (hematocrit), PLT (platelet), PDW (platelet distribution width), RDW (red cell distribution width), TC (total cholesterol) level, TG (triglyceride) level, LDL-C (low-density lipoprotein cholesterol) level, HDL-C (high-density lipoprotein cholesterol) level, very low-density lipoprotein (VLDL) level, and D-dimer level. All the biomarkers data were obtained from the laboratory tests that were closest at the diagnostic time of postoperative DVT.

### Statistical analysis

Continuous data were shown in the form of means and standard deviations (SD). Categorical data were shown in the form of numbers and percentages. Continuous variables were compared with Student *t* test (normal distribution) or Mann-Whitney *U* test (non-normal distribution). Categorical variables were compared with chi-square or Fisher’s exact test. A multivariate logistics regression model was established and a stepwise backward elimination method was utilized to determine independent risk factors correlated with DVT. Variables with *p* < 0.10 were kept in the final model, and the correlation strength was expressed in terms of OR (odds ratio) and 95% CI (95% confidence interval). The statistical test level was *p* < 0.05. Hosmer-Lemeshow (H-L) test was performed to assess the fitting degree of the final model, and *p* > 0.05 indicated eligibility. All data were analyzed in SPSS23.0 (IBM, Armonk, New York, USA).

## Results

A total of 716 patients with isolated patella fractures, who underwent surgical treatment, met the inclusion criteria. There were 425 male and 291 female patients. The average age was 51.3 ± 14.6 years (range,18.0-92.0 years). The average preoperative stay (from injury to operation) was 3.8 ± 2.8 days (range, 0-23.0 days), and the average hospitalization stay was 11.8 ± 5.4 days (range, 2.0-44.0 days). During the period of hospitalization, no one was diagnosed with symptomatic PE (pulmonary embolism). Of the 716 patients, 29 cases were diagnosed with postoperative DVT with the incidence rate of 4.1%. One case (3.4%) developed DVT in bilateral limbs (injured and uninjured). There was DVT located in superficial femoral common vein in 1 case (3.4%), popliteal vein in 6 cases (20.7%), posterior tibial vein in 11 cases (37.9%), and peroneal vein in 11 cases (37.9%). DVT was absent in the anterior tibial vein. The median of the interval between surgery and diagnosis of DVT was 4.0 days (range, 1.0-8.0 days). The results of univariate analysis were presented in Table 1. A total of 11 variables were different between DVT and non-DVT cases. These 11 variables included age (continuous, *p* = 0.002; categorical, *p* = 0.000), hypertension (*p* = 0.008), arrhythmia (*p* = 0.000), chronic lung diseases (*p* = 0.038), preoperative stay (*p* = 0.004), intraoperative blood loss (*p* = 0.015), surgical duration (*p* = 0.000), LDL-C (> 3.37 mmol/L) (*p* = 0.027), HCT (<lower limit) (*p* = 0.021), PLT (> 300 × 10^9^/L) (*p* = 0.010), D-dimer (> 0.3 mg/L) (*p* = 0.007). Variables significantly different in the univariate analysis and the variable of alcohol consumption (*p* = 0.051) were involved in the multivariate model. Six variables were determined to be independent risk factors for DVT. These six variables included age category (> 65 years old), OR, 4.44 (1.34-14.71); arrhythmia, OR, 4.41 (1.20-16.15); intra-operative blood loss, OR, 1.01 (1.00-1.02); preoperative stay (delay of per day), OR, 1.43 (1.15-1.78); surgical duration, OR, 1.04 (1.03-1.06); and LDL-C (> 3.37 mmol/L), OR, 2.98 (1.14-7.76) (Table 2). Hosmer and Lemeshow test revealed good fitness of the final model (*p* = 0.727).

**Table 1:** Univariate analyses of risk factors associated with DVT of lower extremity following isolated patella fracture (continued)

Variables	Non-DVT (*n* = 687) (number (%))	DVT (*n* = 29) (number (%))	*p*
Gender (male)	406 (59.1)	19 (65.5)	0.491
Age (year)	51.0 ± 14.5	59.6 ± 14.6	0.002
18–44	228 (33.2)	6 (20.7)	0.000
45–64	338 (49.2)	9 (31.0)	
65 or older	121 (17.6)	14 (48.3)	
BMI (kg/m^2^)			0.937
18.5–23.9	412 (60.0)	18 (62.1)	
< 18.5	24 (3.5)	0 (0)	
24.0–27.9	185 (26.9)	8 (27.6)	
≥ 28.0	66 (9.6)	3 (10.3)	
Cigarette smoking	90 (13.1)	4 (13.8)	0.784
Alcohol consumption	63 (9.2)	6 (20.7)	0.051
Diabetes mellitus	71 (10.3)	6 (20.7)	0.115
Hypertension	142 (20.7)	12 (41.4)	0.008
Ischemic heart disease	41 (6.0)	3 (10.3)	0.414
Arrhythmia	29 (4.2)	7 (24.1)	0.000
Chronic lung diseases	6 (0.9)	2 (6.9)	0.038
History of cerebral infarction	25 (3.6)	2 (6.9)	0.300
History of any surgery	116 (16.9)	8 (27.6)	0.136
Injury mechanism (high energy)	90 (13.1)	3 (10.3)	0.665
Fracture type (comminuted)	69 (10.0)	6 (20.7)	0.110
Preoperative stay	3.0 ± 1.8	4.8 ± 3.2	0.004
Total hospital stay	11.9 ± 6.6	13.4 ± 6.9	0.238
Anesthesia (general)	32 (4.7)	3 (10.3)	0.164
ASA class			0.174
III and above	33 (4.8)	3 (10.3)	
I and II	654 (95.2)	26 (89.7)	
Intra-operative blood loss	98.5 ± 42.8	126.9 ± 58.4	0.015
Surgical duration	95.5 ± 30.3	139.3 ± 37.8	0.000
Tourniquet use (yes)	553 (80.5)	26 (89.7)	0.219
TP (< 60 g/L)	327 (47.6)	11 (37.9)	0.307
ALB (< 35 g/L)	202 (29.4)	5 (17.2)	0.157
FBG (> 6.1 mmol/L)	209 (30.4)	7 (24.1)	0.470
TC (> 5.2 mmol/L)	53 (7.7)	3 (10.3)	0.489
TG (> 1.7 mmol/L)	149 (21.7)	5 (17.2)	0.568
LDL-C (> 3.37 mmol/L)	180 (26.2)	13 (44.8)	0.027
VLDL (> 0.78 mmol/L)	147 (21.4)	5 (17.2)	0.592
HDL-C (< 1.1 mmol/L)	189 (27.5)	7 (24.1)	0.690
WBC (> 10 × 10^9^/L)	141 (20.5)	5 (17.2)	0.667
NEUT (> 6.3 × 10^9^/L)	248 (36.1)	11 (37.9)	0.084
LYM (< 1.1 × 10^9^/L)	108 (15.7)	5 (17.2)	0.796
RBC (<lower limit)	146 (21.3)	9 (31.0)	0.210
HGB (<lower limit)	148 (21.5)	10 (34.5)	0.100
HCT (<lower limit)	121 (17.6)	10 (34.5)	0.021
PLT (> 300 × 10^9^/L)	42 (6.1)	6 (20.7)	0.010
PDW (> 18.1%)	13 (1.9)	1 (3.4)	0.442
RDW (> 16.5%)	6 (0.9)	0 (0)	0.780
D-dimer (> 0.3 mg/L)	299 (43.5)	20 (69.0)	0.007

*DVT* deep venous thrombosis; *BMI* body mass index; *ASA* American Society of Anesthesiologists; *RBC* red blood cell, reference range: females, 3.5–5.0 × 10^12^/L, males, 4.0–5.5×10^12^/L; *HGB* hemoglobin, reference range: females, 110–150 g/L, males, 120–160 g/L; *FBG* fasting blood glucose; *HCT* hematocrit, 40–50%; *WBC* white blood cell; *NEUT* neutrophile; *LYM* lymphocyte; *PLT* platelet, 100–300 × 10^9^/L; *TP* total protein; *ALB* albumin; *RDW* red cell distribution width; *PDW* platelet distribution width; *TC* total cholesterol; *TG* triglyceride; *LDL-C* low-density lipoprotein cholesterol; *HDL-C* high-density lipoprotein cholesterol; *VLDL* very low-density lipoprotein

**Table 2:** Multivariate analyses of risk factors associated with DVT of lower extremity following isolated patella fracture

Risk factors	OR (95% CI)	*p*
Arrhythmia	4.41 (1.20-16.15)	0.025
Intra-operative blood loss	1.01 (1.00-1.02)	0.023
Surgical duration	1.04 (1.03-1.06)	0.000
Preoperative stay (delay of each day)	1.43 (1.15-1.78)	0.001
LDL-C (> 3.37 mmol/L)	2.98 (1.14-7.76)	0.025
Age category		0.019
18–44	Reference	
45–64 (1)	1.26 (0.39-4.04)	0.700
65 or older (2)	4.44 (1.34-14.71)	0.015

*DVT* deep venous thrombosis; *OR* odds ratio; *CI* confidence interval; *LDL-C* low-density lipoprotein cholesterol

### Treatment of diagnosed postoperative DVT cases

Three of the diagnosed cases (two located in popliteal vein and one in superficial femoral common vein) of DVT were treated with insertion of retrievable IVCF (inferior vena cava filters) combined with anticoagulation therapy. The other diagnosed cases of DVT were treated with intravenous infusion of heparin during hospitalization, and oral warfarin for 3 months after discharge. APTT (activated partial thromboplastin time) was used to monitor the therapeutic level of heparin while INR (international normalized ratio) was used to monitor the therapeutic level of warfarin.

## Discussion

Researchers have focused on the incidences of DVT following surgical treatment of the lower extremity fractures. However, many researchers failed to differentiate specific fracture sites [14, 15]. Studies showed that the incidence of DVT at each anatomic location of the fracture varies greatly [7, 11]. The results demonstrated that the incidence rate of DVT in isolated patella fractures was 4.1%. Jared A et al. studied the incidence rate of postoperative VET (venous thromboembolism) in patients with lower extremity trauma from 2008 to 2016 in the USA [10]. The results showed that the incidence rate of DVT in patients with patella fractures was 0.6% (range, 0.0-1.5%). However, the specific location of the DVTs was not mentioned in the study. In Wang et al.’s study, a small cohort of 59 patients with isolated patella fractures was included [11]. The DVT was categorized into proximal DVT (localized in the popliteal vein or proximally) and distal DVT (localized distal to popliteal vein). The postoperative incidence rate of proximal DVT was 1.7% (1/59) while distal DVT was 23.7% (14/59). In this research, the incidence rates of proximal and distal DVT were 1.0% (7/716) and 3.1% (22/716) respectively. Intermuscular vein blood clot was excluded in this research but was involved as the distal DVT in Wang et al.’s study. This explains the higher incidence of distal DVT in Wang et al.’s study [11].

Both of the above studies indicated that DVT following isolated patella fractures mostly referred to the calf vein (gastrocnemius, soleal, anterior tibial, posterior tibial, and peroneal veins). However, controversy exists on the treatment of isolated calf DVT. The effects of anticoagulation on the morbidity and mortality of calf DVT remain inconclusive [13, 16–19]. Treatment recommendations included monitored observation combined with anticoagulation therapy. Further investigations were needed to determine the ideal treatment option. In this study, three (one in femoral common vein, two in the popliteal vein) of the diagnosed cases of DVT received insertion of retrievable IVCF (inferior vena cava filter) followed by anticoagulation therapy. Intravenous heparin infusion was administered into the other DVT patients during hospitalization period. These DVT patients were also given monitored oral warfarin for 3 months after discharge. None of the cases was diagnosed as symptomatic PE.

The risk of PE and mortality grew greatly due to the complication of DVT in patients who underwent major orthopedic surgeries. Chemical thromboprophylaxis has become a routine procedure after major orthopedic surgeries [20]. However, routine use of chemical prophylaxis in isolated lower extremity fractures is controversial. According to the 9th American College of Chest Physicians Evidence-Based Clinical Practice Guideline, the use of thromboprophylaxis is recommended in high-risk situations such as patients undergoing major joint surgery in hips and knees or hip fracture surgery. However, thromboprophylaxis is not recommended in patients with isolated lower-leg injuries who need leg immobilization. Studies have investigated the impact of chemical prophylaxis on lower extremity fractures [21]. Se-Jun Park et al. performed a study to compare the incidence of thrombus treated with and without thromboprophylaxis in patients with lower extremity fractures [14]. Se-Jun Park et al. concluded that thromboprophylaxis decreases the incidence of postoperative VTE. Zheng et al. also performed a study to determine the influence of chemical thromboprophylaxis on postoperative DVT in patients who underwent foot and ankle fractures [8]. Zheng et al. concluded that it was unnecessary to administer routine chemical thromboprophylaxis in such fractures. There was a controversy over whether routine chemoprophylaxis was necessary for lower limb fractures. Studies have shown that DVT was unpreventable in major orthopedic trauma despite adherence to modern prophylactic protocols [20, 22]. The incidence rate of DVT never decreased despite all patients receiving routine thromboprophylaxis [11]. Routine prophylaxis should be recommended in operative patients with isolated patella fractures.

Identification of risk factors for postoperative DVT in patients who underwent patella fractures is of great significance. In this study, multivariate analysis revealed that age category (age > 65 years old), arrhythmia, intra-operative blood loss, surgical duration, preoperative stay (each day delay in surgery), and LDL-C (> 3.37 mmol/L) are independent risk factors for postoperative DVT in patients with isolated patella fractures. Advanced age correlated with increased occurrence of DVT. However, few studies indicated that there is no correlation between the occurrence of thromboembolic events and age [23]. The vascular system gradually aged as the age increased. Advanced age has been identified to be an independent risk factor for DVT in patients with lower extremity fractures in multiple studies. Lee SY et al. found that the relative risk of DVT was five times higher in 50-69 years old patients while 10 times higher in > 70 years old patients compared with < 49 years old patients in his study [24]. Se-Jun Park et al. also found one independent risk factor for DVT to be patients who are > 60 years old [14]. In this study, patient age was divided into three categories: 18–44 years old, 45–64 years old, and > 65 years old patients. The risk for DVT significantly increased in the category of age > 65 years old, (*p* = 0.015). This was slightly different with the Lee SY et al.’s results [24]. This implied that the effect of increased age on occurrence of DVT was due to aging blood vessels rather than increased age itself. Additionally, there were individual variations in vascular aging.

Studies have analyzed the effect of various comorbidities on the occurrence of DVT. Comorbidities such as hypertension, coronary heart disease, arrhythmia, diabetes mellitus, and chronic lung disease have been reported to be risk factors for DVT in different studies [25]. However, the results from these studies are not entirely consistent with each other. In this study, only arrhythmia was found to be a predictor for DVT in patients who underwent isolated patella fracture surgeries. The relation between comorbidities and occurrence of DVT is very complicated and may be related to individual variations. Blood lipids had been identified to be etiologic factor for DVT in previous studies [26, 27]. LDL-C (> 3.37 mmol/L) was a risk factor for DVT in patients who underwent patella fracture surgeries. A previous study demonstrated that the lower HGB correlated with DVT [28]. However, this association was not confirmed.

Delayed operation prolonged immobilization of the wounded lower extremity. This was one of the main etiological factors for DVT in trauma patients. Smith et al. found that if operation was delayed > 1 day, the daily increment incidence was 14.5% while it was 33.3% if the operation was delayed > 7 days in a prospective study [29]. In this study, the daily increment incidence was 43%. The average preoperative stay was 4.8 days in DVT cases while 3.0 days in non-DVT cases (*p* < 0.001). The reasons for delay in surgery in the institution were mainly as follows: (a) many patients were referred to the hospital from lower-level hospitals, (b) multi-disciplinary consultation and preoperative evaluation in the elderly patient for severe comorbidities, etc.

The surgery-related factors were reported to influence the occurrence of postoperative DVT. A study showed that an increase in surgical duration was closely correlated with an increase in the risk for VTE [30]. This was similar with the result of this study. Surgical duration was identified to be one of the risk factors for DVT in the multivariate model. A longer surgical duration was often accompanied with more intra-operative blood loss. Other surgery-related factors such as anesthesia, ASA class, and tourniquet use were not identified to be risk factors for postoperative DVT in patients with isolated patella fracture [25].

This research had some limitations. First, the research was retrospective which affected the precision of the data. Secondly, some cases were randomly abandoned for incomplete data which could have influenced the results. Thirdly, this was a single-center study; thus, multi-center study is needed in the future. Fourthly, Doppler ultrasound scanning was conducted only in the patients clinically suspected of DVT. Additionally, thrombus monitoring was limited to hospitalization. After discharge, the thrombus was not continuously monitored, which could have underestimated DVT incidence.

## Conclusion

Incidence of postoperative DVT in patients with isolated patella fractures is substantial. More attention should be paid on postoperative DVT prophylaxis in patients with isolated patella fractures. Identification of associated risk factors can help clinicians recognize the risk population, assess the risk of DVT, and develop personalized prophylaxis strategies.

## Data Availability

All the data will be available upon motivated request to the corresponding author of the present paper.

## Abbreviations

DVT: Deep venous thrombosis
VET: Venous thromboembolism
PE: Pulmonary embolism
BMI: Body mass index
EMR: Electronic medical records
ASA: American Society of Anesthesiologists
SD: Standard deviation
OR: Odds ratio
CI: Confidence interval
RBC: Red blood cell
HGB: Hemoglobin
ALB: Albumin
FBG: Fasting blood glucose
HCT: Hematocrit
WBC: White blood cell
NEUT: Neutrophile
LYM: Lymphocyte
PLT: Platelet
TP: Total protein
PDW: Platelet distribution width
RDW: Red cell distribution width
TC: Total cholesterol
TG: Triglyceride
LDL-C: Low-density lipoprotein cholesterol
HDL-C: High-density lipoprotein cholesterol
VLDL: Very low-density lipoprotein
APTT: Activated partial thromboplastin time
INR: International normalized ratio
IVCF: Inferior vena cava filter
